# Nanoparticle-Based Delivery of RNAi Therapeutics: Progress and Challenges

**DOI:** 10.3390/ph6010085

**Published:** 2013-01-16

**Authors:** Jiehua Zhou, Ka-To Shum, John C. Burnett, John J. Rossi

**Affiliations:** 1 Department of Molecular and Cellular Biology, Beckman Research Institute of City of Hope, City of Hope, 1500 East Duarte Rd, Duarte, CA 91010, USA; E-Mails: jzhou@coh.org (J.Z.); kshum@coh.org (K.S.); jburnett@coh.org (J.C.B.); 2 Irell and Manella Graduate School of Biological Sciences, Beckman Research Institute of City of Hope, City of Hope, 1500 East Duarte Rd, Duarte, CA 91010, USA

**Keywords:** small interfering (si)RNA, non-viral vector, multifunctional nanoparticle, targeting delivery, passive and active targeting

## Abstract

RNA interference (RNAi) is an evolutionarily conserved, endogenous process for post-transcriptional regulation of gene expression. Although RNAi therapeutics have recently progressed through the pipeline toward clinical trials, the application of these as ideal, clinical therapeutics requires the development of safe and effective delivery systems. Inspired by the immense progress with nanotechnology in drug delivery, efforts have been dedicated to the development of nanoparticle-based RNAi delivery systems. For example, a precisely engineered, multifunctional nanocarrier with combined passive and active targeting capabilities may address the delivery challenges for the widespread use of RNAi as a therapy. Therefore, in this review, we introduce the major hurdles in achieving efficient RNAi delivery and discuss the current advances in applying nanotechnology-based delivery systems to overcome the delivery hurdles of RNAi therapeutics. In particular, some representative examples of nanoparticle-based delivery formulations for targeted RNAi therapeutics are highlighted.

## 1. Introduction

RNA interference (RNAi) is a cellular mechanism for gene silencing in a sequence-specific manner. RNAi was first observed in plants, later in the worm *Caenorhabditis elegans*, and subsequently in a wide variety of eukaryotic organisms, including mammals [1,2,3]. This process has been demonstrated elegantly in the laboratory to silence certain genes *in vivo* and has contributed significantly to modern scientific and biomedical research [4]. The notion that RNAi could lead to a new class of therapeutics caught the attention of many investigators after its discovery, with the launching of clinical trials for approximately twenty small interfering RNAs (siRNA, a class of double-stranded RNAs of 20-25 base pairs in length that triggers RNAi) or short hairpin RNA (shRNA)-based therapeutics for a variety of human diseases [5,6]. Such RNAi-based therapeutics include siRNA therapeutics for the treatment of age-related macular degeneration (AMD), diabetic macular edema (DME), and respiratory syncytial virus (RSV) (Table 1). Despite the obvious promise, there are several extracellular and intracellular challenges that currently limit the broad use of RNAi in the clinic. For example, Opko Health (previous Acuity Pharmaceuticals) terminated the Phase III trial of bevasiranib for the treatment of AMD in early 2009 because of its poor efficacy in reducing vision loss [7]. Allergan discontinued the Phase II trials of siRNA AGN-745 targeting vascular endothelial growth factor (VEGF) because of a substantial off-target effect [8,9].

Despite these setbacks, some important lessons have been learned from previous trials. Several key hurdles in RNAi delivery must be surmounted in order to realize the clinical translation of RNAi-based therapeutics [10,11]. Investigators in academia and biotech/pharmaceutical industry have made intensive efforts to understand the molecular mechanism of RNAi and develop more advanced RNAi delivery formulations. Currently, viral vectors are one of the major vehicles in gene therapy; however, concerns of potent immunogenicity, insertional mutagenesis and biohazards of viral vectors may present a variety of potential problems to the patient. Non-viral methods could offer certain advantages over viral methods and various innovative non-viral vectors have been vigorously developed to provide a safer and more efficient delivery system.

**Figure 1:**
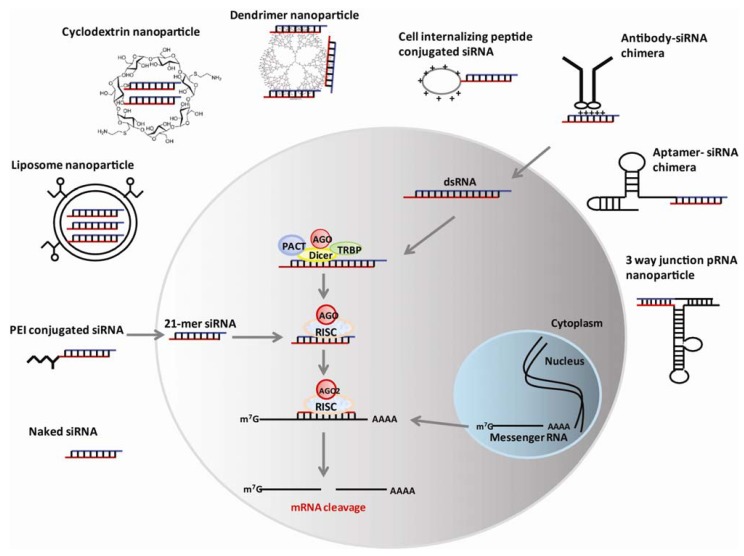
The mechanism and delivery strategies for RNA interference. RNAi therapeutics (e.g., siRNA) can be internalized into the cell via different delivery vehicles. Exogenously introduced long dsRNA are processed into ~21nt siRNA duplex by the Dicer/TRBP complex. Subsequently, siRNA duplexes associate with the Ago proteins and are loaded into the RISC where the passenger (sense) strand is removed and the guide (antisense) strand remains to target mRNA for silencing. The resulting mature RISC complex silences gene expression by cleavage of the mRNA and subsequent degradation, functionally inhibiting the translation of the message.

In particular, the advent of versatile nanotechnology platforms are triggering the development of multifunctional delivery formulations for targeted RNAi therapeutics [12,13,14]. A variety of natural and synthetic nanocarriers (Figure 1 and Table 1), including liposomes, micelles, exosomes, synthetic organic polymers (e.g., polyethylenimine, dendrimer, cyclodextrin), and inorganic materials (e.g., carbon nanotubes, quantum dots, gold nanoparticles) have been developed for siRNA delivery and some of them have entered clinical evaluation [15]. The current review will discuss the major barriers in achieving efficient and safe RNAi delivery and will focus particularly on recent advances in non-viral nanoparticle-based RNAi delivery system.

**Table 1:** Non-viral delivered siRNAs in the clinical pipeline.

Drug name	Disease	Target	Carrier	Phase	Company	Status (Clinicaltrials.gov identifier)
**Local Delivery**						
Bevasiranib	AMD	VEG	Naked siRNA	III	Opko Health Inc.	Terminated (NCT00499590)
AGN-211745 Sirna027	AMD	VEGF	Naked siRNA	II	Allergan/Sirna therapeutics	Terminated (NCT00363714)
PF655	Wet AMD and DME	RTP801	Naked siRNA	II	Quark Pharmaceuticals	Ongoing for DME (NCT01445899); Completed for AMD (NCT00713518)
QPI1007	Non-arteritic ischemic optic neuropathy	Caspase 2	Naked siRNA	I		Ongoing (NCT01064505)
TD101	Pachyonychia congenita	Keratin 6a (K6a) N171K	Naked siRNA	1b	TransDerm	Completed (NCT00716014)
RXI109	Dermal scarring	Connective tissue growth factor	Self-delivering RNAi compound (sd-RxRNA®)	I	RXi Pharmaceuticals	Initiate in 2012
SYL040012	Ocular Hypertension	ADRB2	Naked siRNA	II	Sylentis	Ongoing (NCT01227291)
SYL1001	Dye eye, ocular pain	TRPV1	Naked siRNA	I		Ongoing (NCT01438281)
Excellair	Asthma	Syk kinase	Naked siRNA	II	ZaBeCor	Ongoing
ALN-RSV01	RSV infection	RSV Nucleocapsid “N” gene	Naked siRNA	II	Alnylam Pharmaceuticals	Ongoing (NCT01065935)
siG12D LODER	Pancreatic cancer	KRASG12D	LODER polymer	I	Silenseed	Ongoing (NCT01188785)
**Systemic delivery**						
ALN-TTR	Transthyretin mediated amyloidosis	TTR	Lipid nanoparticles, MC3 lipid	I	Alnylam Pharmaceuticals	Ongoing (NCT01148953, ALN-TTR01; NCT01559077, ALN-TTR02)
ALN-PCS	Hypercholesterolemia	PCSK9	Lipid nanoparticles, MC3 lipid	I		Ongoing (NCT01437059)
ALN-VSP	Liver cancer	KSP and VEGF	Lipid nanoparticles	I		Ongoing (NCT01158079)
TKM-PLK1	Advanced sold tumor	PLK1	Lipid nanoparticles	I	Tekmira Pharmaceuticals	Ongoing (NCT01262235)
KM-Ebola	Zaire Ebola or other hemorrhagic fever viruses infection	RNA polymerase L protein	Lipid nanoparticles, SNALP	I		Ongoing (NCT01518881)
TKM-ApoB (PRO-040201)	Hypercholesterolemia	ApoB	Lipid nanoparticles	I		Terminated (NCT00927459)
Atu027	Advanced Solid tumor	PKN3	Lipid nanoparticles AtuPLEX®	I	Silence Therapeutics	Ongoing (NCT00938574)
QPI-1002 (I5NP)	Delayed Graft Function and Acute Kidney Injury	p53	AtuRNAi chemically modified siRNA	II for Delayed Graft Function I for acute kidney injury	Silence Therapeutics/Quark Pharmaceuticals/ Novartis Pharmaceuticals	Ongoing (NCT00802347)
CALAA-01	Solid tumors	RRM2	Cyclodextrin, PEG and Transferrin	I	Calando Pharmaceuticals	Ongoing (NCT00689065)

## 2. Barriers in Systemic RNAi Delivery

### 2.1. Local Delivery vs. Systemic Delivery

Direct delivery of siRNAs into the cells can be achieved by local administration with eye drops, nasal spray, electronic nebulizer, or endoscopic ultrasound, thereby facilitating a more applicable and noninvasive approach for external or readily accessible diseased organs or tissues (*e.g*., eye, lung, skin).

Initial clinical trials have used intravitreal injection of naked siRNAs into the retina for the treatment of AMD and DME [16]. Other local delivery methods for siRNAs include intranasal route for pulmonary delivery and direct injection into the central nervous system [17,18,19]. Compared with systemic administration, local administration of siRNA encounters fewer cellular barriers and pharmacokinetic concerns [5]. However, many important disease target sites, such as liver and spleen, are neither locally confined nor readily accessible and they can only be reached by systemic administration of siRNAs into the circulatory system [10]. It is therefore imperative to develop safe and efficient systemic RNAi delivery system, particularly for metastatic and malignant cancers, blood diseases and viral infections.

As a typical nucleic acid molecule, siRNA is hydrophilic, sensitive to nuclease degradation, negatively charged, and relatively small. Therefore, these intrinsic physicochemical properties make siRNA extremely poor as an active pharmaceutical ingredient and hence pose serious challenges for successful efficacious siRNA delivery, particularly for systemic administration.

### 2.2. Renal Clearance

The size of an siRNA drug is a principal determinant for biodistribution and bioavailability *in vivo,* as naked siRNA with the average diameter less than 10 nm is rapidly excreted from the blood compartment through renal clearance. When siRNA enters the blood stream by systemic administration, a proper delivery formulation or chemical modification is necessary to increase the retention time of the siRNAs in the circulatory system. Before reaching the target cells, formulated siRNA particles pass through the blood vessel endothelial wall and reach the target organs such as liver, kidney and lymphoid organs [20,21]. Typically, when siRNAs are administered systemically, the amounts of free siRNAs in the kidney are 40-fold greater than in other organs with a circulating half-time lasting only minutes [22].

### 2.3. Vascular Extravasation and Diffusion

After reaching the target tissue, the siRNA drug has to be extravasated from the blood stream into the extracellular matrix [23]. The tumor vasculature has an unique feature of increased leakiness [24]. For example, abnormalities in the tumor vasculature lead to a highly heterogeneous vascular perfusion throughout the tumor, probably facilitating delivery of therapeutics to this region. Typically, macromolecules and nanocarriers (*e.g*., liposomes, nanoparticles) with sizes below 400 nm in diameter can extravasate and accumulate in the “leaky” vasculature of tumor tissue more effectively than in normal tissues *via* passive leakage, thereby increasing the concentration of drugs in tumors and enhancing the therapeutic index [25,26]. This passive extravasation is termed tumor-selective enhanced permeation and retention (EPR) effect [27,28]. Currently, by taking an advantage of the EPR effect, various nanomaterials for *in vitro* or *in vivo* siRNA delivery have been designed and engineered [15]. Many of the nanoparticles are about 100 nm in diameter and exhibit enhanced accumulation around the leaky regions of the tumor vasculature.

Passive diffusion of macromolecules and nanoparticles in the extracellular matrix (ECM) is critical for drug delivery in tumor tissues [29]. Due to the hyperpermeability of the abnormal vasculature and the lack of functional lymphatics, the interstitial fluid pressure (IFP) is elevated in tumor tissue, which reduces convective transport across the vessel wall and into the interstitial space [30]. Therefore, the movement of nanoparticles to the poorly perfused regions of tumors depends primarily on diffusion [29]. Passive targeting requires particles with large diameters, but this simultaneously hinders penetration into the dense collagen matrix of the interstitial space, thereby restricting accumulation of nanoparticles around tumor blood vessels and resulting in less penetration into the tumor parenchyma [31,32]. In this regard, a nanocarrier should be precisely engineered to achieve favorable surface properties and controllable size. For example, Wong *et al*. proposed a multistage nanoparticle delivery system in which nanoparticle is able to change its size to facilitate transport by adapting to each physiological barrier [33]. In this system, the original nanoparticle is 100 nm in diameter, which preferentially extravasates from the leaky regions of the tumor vasculature. After extravasation into tumor tissue, the nanoparticle shrinks to 10 nm, significantly lowering the diffusional hindrance in the interstitial matrix and allowing penetration into the tumor parenchyma.

Because of the low specificity of the EPR-mediated delivery and unpredictable interstitial delivery in different tumors where pore sizes vary greatly, passive targeting may be insufficient for efficient targeting. Therefore, active targeting strategy can significantly facilitate the entry of siRNA drugs into the tumor cells from the extracellular space [34,35]. Decorating siRNAs with active targeting ligands, such as antibodies, peptides, aptamers and small molecules, allows accumulation of siRNAs in the targeted tissue and promotes the cell-specific binding and uptake *via* receptor-mediated endocytosis. For example, Calando Pharmaceuticals designed a polymer based nanoparticle coated with human transferrin, which can specifically bind to transferrin receptor expressed on tumor cells for the treatment of metastatic melanoma [36,37].

### 2.4. Cellular Uptake and Endosomal Escape

Cellular internalization of non-viral, synthetic vectors involves an endocytosis pathway, which can be divided into four different subtypes, 1) clathrin-mediated endocytosis; 2) caveolae-mediated endocytosis; 3) clathrin- and caveolae-independent endocytosis; and 4) phagocytosis and macropino-cytosis [38,39,40]. Targeting ligands that recognize specific antigens on the surface of target cells could enhance intracellular uptake by binding to cell surface receptors. Following cellular uptake, the endocytic vesicles generated by the nanocarrier-siRNA system travel along microtubes and subsequently fuse with early endosomes. Later on, they mature into late endosomes (pH 5-6) and finally enter into lysosomes, which are the last compartment of the endocytic pathway [41]. The lysosomes are further acidified (pH ~4.5) and contain various nucleases. Hence, it is not surprising that a large portion of the siRNAs will be hydrolyzed after a long journey to reach target mRNAs. For efficient gene silencing, siRNA has to escape from the endosomes before it is subjected to degradation in the lysosomal compartments. Endosomal escape is thought to be a major barrier for efficient siRNA delivery [42]. In this regard, different nanocarriers have been developed to improve the endosomal escape of siRNAs into the cytoplasm. For example, many cationic polymers (e.g., PEI or dendrimer) have a strong buffering capacity over a range of pH between 5 and 7 that can mediate efficient siRNA delivery in various cell lines and animal models. In this case, the acidic lysosomal environment can result in protonation of amine groups in the PEI or dendrimer, thereby causing osmotic swelling and finally vacuole disruption accompanied by endosomal release of the polymer and its siRNA cargo [43,44]. Collectively, this phenomenon is termed the “proton-sponge” effect. Some pH-sensitive lipoplexes or polyplexes have been developed for efficient endosomal escape and intracellular trafficking of the siRNAs [11].

Additionally, siRNA delivery can be improved considerably by the use of endosome-disruption agents such as chloroquine (CQ) [45], Ca^2+^ [46], sucrose [47], and photosensitizers [48] (photo-chemical internalization (PCI) treatment). Such endosome-disruption treatments can facilitate the escape of siRNAs from endosomal compartments. Recently, some photosensitizing carriers were also employed for photoinduced RNAi activity [49,50]. For example, CLIP-RNAi (CPP-linked RBP-mediated RNA internalization and photo-induced RNAi) was developed as a photoinducible RNAi method using photosensitizing carrier molecules [51,52,53]. This system consists of a cell-penetrating peptide (CPP), RNA-binding protein (RBP) and a fluorescent dye as a photosensitizer. Most recently, Braun et al. described a new approach using a combination of laser-dependent desorption of thiolated siRNAs from gold nanoparticles (AuNPs) and siRNA/AuNP release from endosomes [54]. Their results demonstrated temporally and spatially controlled gene silencing *in vitro* through the AuNP-activated release of siRNAs using a pulsed near-infrared (NIR) laser.

### 2.5. Cytoplasmic Location and RISC Loading of siRNAs

Once siRNAs are released into cytoplasm, one of the strands (guide) of siRNAs is loaded into RISC, which contains different argonaute (Ago) family proteins [55,56,57]. Once loaded into an Ago protein, the guide stand of the siRNA identifies a complementary target mRNA sequence for the sequence-specific degradation (Figure 1), while the other strand (passenger) of siRNA is either rejected and/or degraded [58]. It has been demonstrated that RISC is concentrated in specific cytoplasmic locations rather than randomly distributed in the cytoplasm [59,60,61]. Additionally, Ago2, which is capable of catalyzing mRNA degradation, is localized in cytoplasmic processing bodies (P-bodies), where the mRNA is deadenylated and destroyed [62,63]. In the current nanocarrier delivery system, siRNAs are released into cytoplasm seemingly at no particular location. Further mechanistic studies about the intercellular fate of siRNA, such as RISC loading, Dicer processing, half-life of siRNA and strand selectivity are necessary to increase the delivery efficiency of siRNAs.

## 3. Nanotechnology-Based RNAi Delivery

Over the past decade, the emergence of nanotechnology has proven to have a significant impact on drug delivery. A nanotechnology approach to drug delivery focuses on developing nanoscale particles (1-1,000 nm) to deliver drugs to a specific site, extend their bioavailability, improve their biodistribution, reduce their immunogenicity and toxicity and improve their efficacy [64,65,66]. Several nanocarriers have been successfully formulated to engineer anti-cancer chemotherapeutics, including paclitaxel, doxorubicin and dexamethasone that are available on the market [65]. In the following section, we overview the recent advances in nanotechnology development for siRNA delivery (Figure 1).

### 3.1. Liposome-Based Nanoparticles

Liposomes are probably the most extensively studied materials for drug delivery [67]. Several liposomes have proven safe and efficient for delivering small molecule drugs in patients. For instance, doxorubicin liposomal (Doxil; Orthobiotech) has received FDA approval for treatment of HIV-related Kaposi's sarcoma, breast cancer, ovarian cancer, and other solid tumors [68]. Liposomal amphotericin B (Ambisome; Gilead) is an approved antibiotic for the treatment of serious fungal infections [69]. For nucleic acid delivery, many of the lipid-based delivery vehicles self-assemble with siRNAs through electrostatic interactions with charged amines, generating multimellar lipoplexes with positively charged lipids and negatively charged siRNAs [70]. Currently, various lipid compositions, including Lipofectamine 2000 (Life Technologies), Oligofectamine (Life Technologies) and TransIT-2020 (Mirus Bio), have been used routinely in the laboratory to transfect nucleic acids into cells. However, their uses *in vivo* are limited owing to toxicological concerns and poor colloidal stability [71].

In the design of liposome composition, there are two varieties, cationic and anionic. Cationic liposomes, however, are generally more favorable due to their higher transfection efficiencies [72]. For example, 1,2-dioleoyl-3-trimethylammoniumpropane (DOTAP), Transfectam (Promega) and 98N12-5 are commonly used building blocks for liposome based delivery system [38,73]. When cationic lipids combine with anionic siRNAs, 70-nm diameter amorphous particles known as lipoplexes are formed that subsequently facilitate the release of the siRNAs into cells by formation of neutral ion pairs between cationic liposome carriers and the more anionic cellular plasma membrane. Typically, most siRNAs enter cells *via* endocytosis, suggesting that an endosome escape mechanism is required for efficient gene silencing. In fact, efficient siRNA release from the endosome represents the most critical challenge in delivery as endosome internalization leads to degradation of the siRNAs. Some amine-based materials such as polyethylenimine (PEI) and β-amino ester were reported to promote endosome escape *via* the proton sponge effect, which induces the rupture of the endosome to release its siRNAs to the cytoplasm [74]. Also, pH sensitive bonds and fusogenic peptides were designed so the lipid carriers were destabilized to promote release of the siRNAs in the acidic endosome environment [38,75].

More recently, some lipid-based carriers have begun to be evaluated in the clinical development pipeline. Tekmira Pharmaceuticals, in partnership with Alnylam Pharmaceuticals, has developed a specialized liposome nanoparticle termed a Stable Nucleic Acid Lipid Particle (SNALP) that represents one of the major achievements in systemic siRNA delivery to date [76]. SNALPs are poly-ethylene glycol (PEG)-conjugated liposomes comprised of siRNA encapsulated inside a lipid bilayer of cationic lipids, neutral lipids and PEG-lipid fusion regulators. Comparing with conventional liposomal siRNA complexes, SNALP formulation has a longer half-life in plasma and liver. Two SNALP-formulated siRNA drugs were designed to treat high levels of blood cholesterol or hypercholesterolemia, though TKM-ApoB (Tekmira) targeted ApoB [5]; while ALN-PCS (Alnylam) targeted proprotein convertase subtilisn/kexin type 9 (PCSK9) [77]. Both drugs were tested in Phase 1 clinical trials and were determined to be safe and well tolerated with no serious adverse events associated with drug administration. Additionally, Alnylam Pharmaceuticals is currently developing two SNALP encapsulated siRNA drugs that targeted the transthyretin (TTR) gene to treat TTR-mediated amyloidosis (ATTR). In the Phase 1 trials of ALN-TTR01, it was demonstrated that administration of ALN-TTR01 exhibits a statistically significant dose-dependent reduction in serum TTR protein levels in ATTR patients. More recently, ALN-TTR02 comprises siRNAs targeting the same gene but is encapsulated by the second-generation proprietary nanoparticles called MC3 lipids (http://www.alnylam.com). A Phase 1 study of ALN-TTR02 has recently been initiated to evaluate its safety, tolerability and clinical activity in healthy volunteers (http://www.alnylam.com). A similar lipid formulation (ALN-VSP) is in Phase 1 clinical trials, delivering siRNAs against two important cancer genes, kinesin spindle protein (KSP) and vascular endothelial growth factor (VEGF) for the treatment of liver cancers. This dual-targeting strategy demonstrated strong evidence of anti-tumor activity in advanced malignancy patients. Importantly, the two siRNAs targeting VEGF and KSP in ALN-VSP were detected in nearly all of the biopsy samples [78].

Using a similar approach to SNALP, other liposome-based nanoparticle designs are being evaluated in multiple Phase 1 clinical trials. Silence Therapeutics developed the proprietary AtuPLEX system to deliver 2'-O-methyl-modified siRNAs (Atu027) against protein kinase 3 (PKN3) to the vascular endothelium for the treatment of solid tumors [79]. Early clinical data revealed that Atu027 exhibits an excellent safety profile and helps to stabilize the diseases of some patients in advanced stages of disease [80].

### 3.2. Cationic Dendrimers

Dendrimers are a highly branched synthetic polymers centered around an inner core (~100 nm). As an example, some dendrimers consists of polyamidoamine (PAMAM) synthesized in an algorithmic step-by-step fashion; every repeated branched layer represents a higher generation molecule. Compared to other linear polymers, dendrimers with spherical shape have a well-defined size and chemical structure, and their surface functional groups can be engineered for various applications [81]. Modified PAMAM dendrimers with surface amino groups conjugated to folic acids as targeting vehicles have been used for delivery of the anticancer small molecule methotrexate, demonstrating a better therapeutic index than free drug in animal models [82]. Given the high density of positive charges on the surface, dendrimers are also attractive for delivery of negatively charged plasmid DNA, antisense oligonucleotides, and siRNAs [83,84,85,86]. These nucleic acids can be loaded by surface adsorption or interior encapsulation, thereby protecting them from serum degradation and triggering immune response. To form a stable siRNA-dendrimer nanoparticle, the size-to-charge ratio of dendrimer formulation should be deliberately adjusted so as to provide sufficient electrostatic interactions to form a stable complex while not impeding the release of siRNAs from the endosome into the cytosol [87]. The immunogenicity and toxicity of dendrimers are associated with the surface charge [83], though cationic PAMAM dendrimers are generally more cytotoxic than anionic PAMAM dendrimers.

Recently, our group has reported on the generation 5 (G5) dendrimer for functional delivery of siRNAs that inhibit HIV infection and replication by targeting HIV genes *tat* and *rev* and host dependency factors CD4 and Transportin-3 (TNPO3) [88]. The G5 dendrimer-siRNA complexes demonstrated effective inhibition of HIV-1 replication in T lymphocytes *in vitro* and in a humanized mouse model. In another study, dendrimers were further modified with magnetofluorescent nanoworms to form “dendriworms” to increase the siRNA loading capacity [89]. When the dendriworms carrying siRNAs were added to human glioblastoma cells, this siRNA-dendrimer complex rapidly internalized into the cells and then escaped into the cytosol [89]. The delivered siRNAs were able to silence expression of the targeted gene *in vivo.*

For targeted delivery, dendrimer can be easily conjugated with one or multiple targeting ligands. For example, the 9-mer Luteinizing Hormone-Releasing Hormones (LHRH) peptide was conjugated to PAMAM dendrimers, whose internal amino group was quaternalized for siRNA loading [90]. The cellular uptake was observed to be dependent on the targeting peptide. Similarly, a 53-mer epidermal growth factor (EGF) peptide was grafted with generation 4 (G4) PAMAM dendrimers for siRNA delivery [91].

### 3.3. Cyclodextrin Polymers

Cyclodextrin polymers have received considerable attention as two cyclodextrin based nano-drugs are in clinical trials [92,93]. Cyclodextrins possess defined geometric (~70 nm) and cationic structural characteristics that offer advantages for cationic siRNA payloads to form inclusion complexes. Additionally, each cyclodextrin molecule may contain covalently bound polyethylene glycol (PEG), which acts to stabilize the nanoparticle and avoid nonspecific interaction with blood and extracellular elements under physiological conditions. A variety of targeting agents that recognize cell surface antigens can be covalently attached to the surface PEG modifier.

IT-101 (Calando Pharmaceuticals) is designed for delivery of an anti-cancer drug, camptothecin [94], and while CALAA-01 (Calando) is designed for delivery of siRNAs [36]. Cyclodextrins—the primary carriers for these drugs—are natural cyclic sugars composed of 6(α-CD), 7(β-CD) or 8(γ-CD) D-(+)-glucose units linked by α-1,4 linkages [93]. In CALAA-01, transferrin, which is a blood plasma protein for iron delivery, is employed as a cell internalizing agent since many types of cancer cells have been shown to overexpress transferrin receptors, thereby enabling the uptake of cyclodextrin-siRNA nanoparticles by the cell of interest. The cyclodextrin–siRNA nanoparticle (CALAA-01, Calando Pharmaceuticals) has entered a Phase 1b clinical trial, which is the first-in-human study involving systemic siRNA administration to patients (NCT00689065) [95]. In this nanoparticle delivery system, the siRNA is directed against the M2 subunit of ribonucleotide reductase, which is a critical enzyme in the proliferation of cancer cells. The drug safety of CALAA-01 is currently being assessed in Phase 1b studies in patients with solid organ tumors refractory to treatment (NCT00689065) [95]. Early data from this study showed that this delivery system was able to localize in tumor cells of melanoma patients in a dose-dependent manner and revealed the first proof for an RNAi gene silencing mechanism in humans by a modified 5' rapid amplification of cDNA ends (RACE) assay [37].

### 3.4. Polyethyleneimine (PEI)

Polyethyleneimine (PEI) is a synthetic biocompatible polymer that is available in linear and branched forms with a wide range of molecular weights. Owing to its rich proton-accepting amino group contents, PEI is well known to exert a proton sponge effect to induce endosomal release of siRNA into the cytosol [83]. Hence, PEI is preferentially utilized to complex with other nanoparticles for siRNA delivery [96].

Intradigm (now merged with Silence Therapeutics) links PEI with a three-amino acid cell penetrating peptide (Arg-Gly-Asp) to deliver anti-VEGF siRNAs for the treatment of angiogenesis [97]. The anti-VEGF siRNA nanoparticles exhibited inhibition of VEGF production and demonstrated desirable anti-tumor effects in an animal model, while nanoparticle complexed with control siRNAs did not show inhibitory effects [97,98]. In a similar active targeting approach, several targeting molecules, such as folate, galactose and pulluan, were also reported in the targeted delivery of PEI/siRNA nanoparticles [96,99,100]. However, it is noted that PEI might induce cytotoxicity due to the polycationic nature, leading to membrane damage and activation of the innate immune system [101].

### 3.5. Mesoporous Silica Nanoparticles

Mesoporous silica nanoparticles (MSNs) are another important inorganic material that has long been used for small molecule drug delivery. The large surface area of the pores allows the particles to be filled with large amount of drugs, which will subsequently be taken up by cells through endocytosis. MSNs are formulated by reacting tetraethyl orthosilicate with a template made of micellar rods. The surface of MSNs can be chemically modified with various functional groups for targeting purposes. A number of MSN based systems have been reported to deliver siRNAs for cancer therapy. In one study, a MSN containing PEI and functionalized fusogenic peptide (denoted as M-MSN_siRNA@PEI-KALA) was described to be highly effective for silencing gene expression both *in vitro* and *in vivo* [102]. The assembly of this nanoparticle started with the encapsulation of siRNAs within the pore of MSN, followed by the conjugation of PEI on the surface of the nanoparticles and the coating of KALA peptides. It was shown that the nanoparticles were efficiently internalized into cells and subsequently escaped from the endosome to release the loaded siRNAs into the cytoplasm. Intra–tumoral injection of M-MSN_siRNA@PEI-KALA significantly inhibited tumor growth with negligible cytotoxic effect. Furthermore, because of the large capacity of MSNs, it was reported that cancer chemotherapeutics, such as doxorubicin, can be loaded together with Bcl2-siRNAs into MSN for enhanced efficacy of chemotherapy [103]. It was observed that both Bcl2-siRNA and Dox were released into cells. The Bcl2-siRNA effectively silenced the multi-drug resistant pump of Bcl2 gene, which subsequently enhanced the effect of Dox [103].

### 3.6. Protein or Peptide-Based Nanoparticles

Owing to the specificity of antibodies, it is rational to make use of antibodies to selectively deliver therapeutic payloads into diseased cells [84]. For example, Mylotarg® (gemtuzumab) is a currently FDA-approved monoclonal antibody against CD33 for the selective delivery of small molecule chemotherapeutics to acute myeloid leukemia (AML) cancer cells that overexpress CD33 [104]. Similarly, therapeutic siRNAs can be conjugated to an antibody through a carrier *via* electrostatic association. The antibody can be biologically generated to bind to antigens expressed on the surface of target cells. Once the antibody-siRNA conjugates specifically locate and bind to target cells, the complexes become internalized with the antigen through clathrin mediated-endocytosis and subsequently siRNAs are released to cytoplasm to execute RNAi.

Antibodies can also be reduced in size by only using the antigen recognition site. A single-chain variable fragment antibody (scFv) can be constructed and fused to a protamine fragment for selective siRNA delivery. Examples of antigens that have been adopted for targeted delivery include HIV-1 gp160, Her2 and ErbB2 [105,106,107]. In the study of the gp160 antibody-mediated siRNA delivery, the siRNAs targeting the HIV-1 *gag* gene were complexed to a protamine-gp160 antibody fusion protein [106]. The role of protamine acts as a polycation that binds to approximately six siRNA molecules per fusion protein by charge interactions and forms a highly condensed nanoparticle. These antibody-siRNA complexes were specifically taken up by cells infected with HIV-1 and showed significant inhibition of HIV replication in infected primary T lymphocytes [106]. Recently, a similar strategy using a fusion of Her2 antibody and protamine loaded with Plk1 siRNAs was shown to suppress proliferation of Her2 positive breast cancer cells and primary human cancers in orthotropic breast cancer models [105]. Systemically injected Her2ab/Plk1 siRNA nanoparticles were localized to Her2 expressing cells in xenografts and persisted for more than 72 hours, leading to Plk1 gene silencing and tumor cell apoptosis [105].

Alternatively, the protamine fusion can be substituted with a 9-amino acid poly-(D)-arginine peptide, but the siRNA loading capacity is dramatically reduced compared with the protamine fusion approach. Anti-HIV siRNAs against the host CCR5 co-receptor and HIV genes *vif* and *tat* were conjugated with poly-(D)-arginine and CD7 antibody to facilitate binding and internalization into CD7-expressing human T lymphocytes [108]. Moreover, systemic injection of these nanoparticles showed robust protection of T lymphocytes from HIV infection in humanized mice, and the fusion protein-siRNA complex did not cause toxicity in transfected cells or trigger interferon responses [108].

Cell penetrating peptides (CPPs) are short chains of acidic amino acids that have been evaluated as siRNA delivery agents [109,110,111,112]. The idea of using positively charged peptides as delivery vehicles for siRNA is similar to the strategy of cationic liposomes, as these short peptides can spontaneously form complexes with siRNAs by electrostatic interactions. Subsequently, the complexes can interact with anionic cell membranes to facilitate release of siRNAs in cells.

Tat-(48-60) (GRKKKRRQRRRPPQ) and penetratin® (RQIKIWFQNRRMKWKKC) are the most extensively studied cell penetrating peptides derived from the HIV-1 trans-activator of transcription protein and the third helix of the homeodomain of the antennapedia protein, respectively [113,114]. These peptides have demonstrated efficient gene knockdown when the 3' end of the sense strand siRNA is conjugated with the cell penetrating peptides. To facilitate efficient incorporation into the RISC, the siRNAs and cell penetrating peptides may be linked by a disulfide bond so that each component can separate in the reducing environment of the cytosol [110]. Interestingly, when the peptides are attached to the antisense strand of siRNAs, the efficiency of gene knockdown is significantly reduced, probably due to disruption of RISC loading [115]. Traversa Therapeutics and Sanofi-Aventis are validating and developing the Tat-based cell penetrating peptide system for siRNA delivery.

In addition to direct conjugation with siRNAs, cell penetrating peptides can be coated on the surface of other delivery vehicles to refine their properties. For example, Tat peptide conjugated PEGylated liposomes were prepared and exhibited better cellular intake than PEGylated liposomes alone [83,116]. Histidine-lysine (HK) peptides are also designed to enhance endosomal disruption ability by the proton sponge effect of the nanoparticles [117].

### 3.7. Nucleic acid Aptamer–Based Nanoparticles

Akin to antibodies, there has been a major interest in the application of aptamers for targeted siRNA delivery [118]. Aptamers are single-stranded nucleic acids that are evolved *in vitro* by an iterative selection process called Systematic Evolution of Ligands by Exponential Enrichment (SELEX) [119]. Aptamers can specifically recognize and bind to their cognate targets through their well-defined stable three dimensional structure [120]. The use of aptamers can serve dual functions as agents that can target cell receptors and as vehicles to deliver siRNA cargoes to specific cells based on surface markers [121,122,123]. The cell-specific aptamers can be conjugated to therapeutic agents or nanocarriers for targeting purpose. Nanocarriers that have large surface areas and interior cavities can assemble multiple aptamers and various drugs molecules, thereby increasing their binding affinity and loading capacity. Currently, a number of aptamer-functionalized nanocarriers have been generated for targeting RNAi [124,125]. For example, Zhao *et al*. formulated a non-covalent nanocomplex that specifically silenced anaplastic lymphoma kinase (ALK) gene expression and induced growth arrest and apoptosis in CD30-expressing anaplastic large cell lymphoma (ALCL) cells. The complexes with average diameter ~140 nm are formed by direct mixing of individual siRNAs, aptamers and PEI [126].

Aptamers can be synthesized chemically and are amenable to chemical modification. During chemical synthesis of aptamer, various functional groups, such as amino group (-NH_2_) or thiol group (‑SH), are readily incorporated into the nucleic acid molecules. For example, a prostate-specific membrane antigen (PSAM) RNA aptamer containing a 5’-NH_2_ group was chemically conjugated with a branched polyethyleneimine-grafted polyethylene glycol polymer (PEI-PEG) that serves as a vehicle for shRNA delivery [127]. The PEG linker served as a spacer to separate positively charged PEI from negatively charged aptamers, thereby minimizing their potential interactions. Similarly, a 3'-SH modified anti-PSMA RNA aptamer (A10-3.2) was covalently coupled to the surface of PAMAM dendrimer using a PEG linker. The resulting PSMA aptamer-PEG-dendrimer effectively delivered miR-15a and miR-16-1 to prostate cancer cells overexpresing PSMA, resulting in cell apoptosis. Recently, a multifunctional nanoparticle system containing a PEI-coated quantum dot was used to absorb siRNAs *via* non-covalent electrostatic interactions. PSMA aptamer containing a thiol group was conjugated to the siRNAs *via* thiol-disulfide exchange [128]. The resulting PSMA aptamer-S-S-siRNA chimeras/PEI-QD nanoparticles were prone to induce the proton sponge effect that provided improved selective gene silencing activity over non-targeted nanoparticles.

### 3.8. Bacteriophage phi29 Packaging RNA (pNRA)–Based Nanoparticles

Bacteriophage pRNA is a key component in the Phi29 phage packaging motor that gears the viral genome DNA into the viral capsid. pRNA, which is composed exclusively of RNA, is expressed as identical monomers, but can be engineered into dimers, trimers, or up to hexamers via the loop-loop interactions under ambient conditions [129,130]. This feature of forming self-assembled complexes makes pRNA an attractive building block for bottom-up assembly of RNA nanostructures. Each pRNA monomer contains two domains: 1) an interlocking domain and 2) a helical domain. Both domains fold independently. The modification of the helical domain of pRNA (*e.g.,* substitution of a helical domain with an siRNA sequence) does not disturb its folding, structure and intermolecular interactions of the multimer. The pRNA nanostructure can be fabricated to deliver therapeutic siRNAs specifically into diseased cells. For example, targeting molecules, such as folate ligands, were conjugated to the helical domain of one pRNA subunit and then dimerized with another pRNA subunit encoding a therapeutic siRNA that targeted the anti-apoptotic factor survivin gene [131]. Once assembled, these pRNA nanoparticles, ranging from 10 nm to 50 nm, were optimal for cellular intake and were efficiently internalized by cancer cells overexpressing folate receptors. Pre-clinical animal studies also showed that the pRNA nanoparticles only targeted folate expressing cancer cells and promoted apoptosis in xenografts [131,132]. The pRNA/siRNA chimera nanoparticles have also been engineered for targeted delivery of *tat/rev* siRNAs and MT-IIA siRNAs for the treatment of HIV and ovarian cancer respectively [131,133].

Recently, the three-way junction (3WJ) of the pRNA was exploited to carry functional RNAs (e.g., siRNA, aptamers, and receptor ligands like folate) at the end of each junction [134]. When three oligomers containing functional modules were mixed together, a thermodynamically stable tripartite nanostructure was formed and more importantly, the functionalities of the RNA modules remained independent, suggesting that the 3WJ domain of pRNA can serve as a nano-platform for the construction of RNA nanoparticles for targeted siRNA delivery to specific cells for the treatment of diseases [134].

## 4. Conclusions and Future Prospects

Since the first publication of RNAi in 1998, this technology has already advanced rapidly from the laboratory bench to the early or mid stage clinical trials. Although several RNAi-based drugs indicate strong promise in clinical applications, occasional recent frustrations in clinical trials have tempered the excitement and have triggered extensive efforts to surmount these key hurdles. Cytoplasmic delivery of siRNAs is one of the most important limitations. As described in Section 2, systemic RNAi delivery involves multi-step processes and endosomal escape is the most challenging bottleneck of translation of RNAi therapeutics. Therefore, it is not surprising that even slight inefficiencies at any particular stage would ultimately lead to a marginal or no gene silencing activity.

Nanotechnology offers an assortment of versatile targeted delivery platforms for RNAi therapeutics. A precisely engineered, multifunctional nanocarrier with combined passive and active targeting capabilities may address the delivery challenge to the widespread use of RNAi as a therapy. Different nanotechnology platforms have their inherent niche and function differently by various routes of delivery (e.g. local *vs.* systemic) that subsequently affect the disease type (Table 1). For example, naked siRNAs, which are rapidly degraded in biological serum, are confined to easily accessible organs; the biodistribution of lipid particles (SNALPs) and dendrimers are more suitable for liver disease by systemic delivery; and aptamer-siRNA chimera and pRNA nanoparticles are multivalent that are suitable for viral diseases. Although SNALPs appear to be the most promising approach in the clinical development pipeline, it is expected that other nanotechnology platforms will display advantages in other disease areas.

To effectively translate preclinical proof-of-concept to clinical efficacy, the following developments must be achieved to advance the field of RNAi therapeutics, 1) optimization of gene silencing activity of RNAi agents with increased nuclease resistance and reduced immune activation; 2) discovery of proper delivery formulation with prolonged circulation time and enhanced biodistribution; 3) specific tissue and cellular uptake; 4) efficient endosomal release of siRNA and incorporation of siRNA into the multi-protein RNA-induced silencing complex (RISC); and 5) elucidation of RISC loading and Ago2 function. A better understanding of intracellular fate of siRNA-nanocarriers will provide more rational rules for designing and optimizing an ideal siRNA-nanocarrier delivery system.
